# Highly Sensitive Adsorption and Detection of Iodide in Aqueous Solution by a Post-Synthesized Zirconium-Organic Framework

**DOI:** 10.3390/molecules27238547

**Published:** 2022-12-04

**Authors:** Jun Zhang, Shanli Yang, Lang Shao, Yiming Ren, Jiaolai Jiang, Huaisheng Wang, Hao Tang, Hui Deng, Tifeng Xia

**Affiliations:** Institute of Materials, China Academy of Engineering Physics, Huafeng Village, Mianyang 621907, China

**Keywords:** metal-organic frameworks (MOFs), post-synthesis, iodide ions, removal, sensing

## Abstract

Effective methods of detection and removal of iodide ions (I^−^) from radioactive wastewater are urgently needed and developing them remains a great challenge. In this work, an Ag^+^ decorated stable nano-MOF UiO-66-(COOH)_2_ was developed for the I^−^ to simultaneously capture and sense in aqueous solution. Due to the uncoordinated carboxylate groups on the UiO-66-(COOH)_2_ framework, Ag^+^ was successfully incorporated into the MOF and enhanced the intrinsic fluorescence of MOF. After adding iodide ions, Ag^+^ would be produced, following the formation of AgI. As a result, Ag^+^@UiO-66-(COOH)_2_ can be utilized for the removal of I^−^ in aqueous solution, even in the presence of other common ionic ions (NO_2_^−^, NO_3_^−^, F^−^, SO_4_^2−^). The removal capacity as high as 235.5 mg/g was calculated by Langmuir model; moreover, the fluorescence of Ag^+^@UiO-66-(COOH)_2_ gradually decreases with the deposition of AgI, which can be quantitatively depicted by a linear equation. The limit of detection toward I^−^ is calculated to be 0.58 ppm.

## 1. Introduction

Among efforts to balance the increasing energy demand of human society and sustainable development, nuclear energy has raised more and more attention as a zero-emission-based source of clean energy. Currently, nuclear power provides ~11% of the world’s electricity and it is estimated to rise substantially to around 80%, to a massive ~715 GW(e)/annum in nuclear energy by the year 2050 [1,2]; however, nuclear energy generated from atom fission produces several harmful radioactive isotopes, among which both radioactive ^129^I and ^131^I are regarded as extremely dangerous due to the large half-life and high volatility of ^129^I and severe metabolic processes interference of ^131^I [3,4,5]; besides, accidental release of radioactive iodine isotopes during an accident at a nuclear reactor holds a serious threat to the environment and human health [6]. For example, high levels of radioactive iodine (^131^I) have been found in the groundwater at the devastated nuclear facility after the nuclear accident at Fukushima [7,8]. In addition, ^131^I is widely utilized for the treatment of hyperthyroidism and thyroid cancer, while ^125^I is a good assistant for thyroid scanning and radioimmunoassay [9]. Overall, the wastes that contain radioactive iodine should be properly stored and disposed of to guarantee public safety [10,11]; therefore, it is undoubted that the development of effective methods for capture and sensing of radioactive iodine from wastewater is of great practical significance.

In environments with a pH of about 4–10, iodine mainly exists as iodide. In the nuclear accident, radioactive iodine was released primarily in the form of iodide, which was found as the dominant form in the marine environment [12,13]. Accordingly, several adsorbents for iodide in aqueous solution have been studied in the past few decades, including hydrotalcite [14], nanofibers [15], Cu/Ag-based material [16,17], bismuth oxide [13,18,19], porous organic polymers [3,20], and metal–organic frameworks (MOFs) [21,22]. Among these materials, MOFs which possess organic–inorganic hybrid features along with porous structures are considered to be promising candidates as scavengers for various environmental pollutants [23,24,25,26,27]. Due to their adjustable channel chemical environment and post-synthetic friendly character, MOFs have been developed as iodide adsorbents through the modification of pore environments or decorating of I^−^ trap moieties, such as Ag^+^ [21,28,29,30] and bismuth [31], which show excellent adsorbent capacity for iodide in aqueous solution. It is not difficult to see that the timely determination of the concentration of iodide is also important after the adsorption process. In this regard, an adsorbent combined with a sensor should be practical. A few methods of sensing iodide are reported in the literature. Trace levels of iodide were usually detected with spectroscopy, which suffers from the need for frequent calibration and the bulky nature of the devices. For iodide sensors at ppm levels, various strategies have been developed, including luminescent sensors and electronic sensors [32,33,34]. These methods demonstrated promising application potential in real-time outdoors sensing, which is very suitable for monitoring environmental safety. MOFs have also been developed as fluorescent sensors in the past two decades [35,36,37,38,39]. Based on a MOF, an iodide adsorbent and sensor might be realized simultaneously. 

**Scheme 1:**
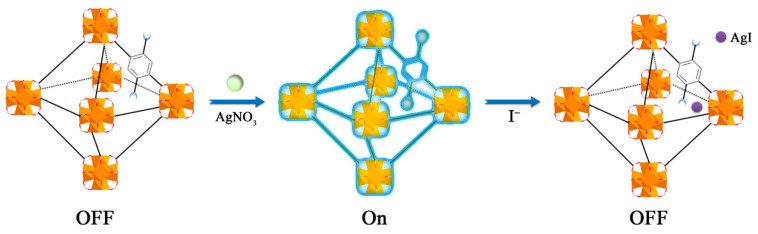
Illustration of the structure of UiO-66-(COOH)_2_ and Ag^+^@UiO-66-(COOH)_2_ as well as its iodide removal and off–on–off luminescent sensing mechanism.

Bearing these considerations in mind, we synthesized a water-stable UiO-66-(COOH)_2_ modified by Ag^+^ for the adsorption and sensing of iodide in aqueous solution. As a proof-of-concept demonstration (Scheme 1), the resulted Ag^+^@UiO-66-(COOH)_2_ was applied to capture iodide from aqueous solution, and afterwards the iodide concentrations can be determined by this material. To the best of our knowledge, this is the first time the iodide in aqueous solution was removed and measured simultaneously based on MOF material. As expected, Ag^+^@UiO-66-(COOH)_2_ exhibits excellent iodide adsorption capacity and ability to sense iodide, which is mainly due to the formation of AgI. Overall, our work demonstrates Ag^+^@UiO-66-(COOH)_2_ can serve as an effective iodide adsorbent and sensor and further suggests the wide application of MOF-based integrated devices. 

## 2. Results and Discussion

### 2.1. Crystal Synthesis and Characterization

**Figure 1:**
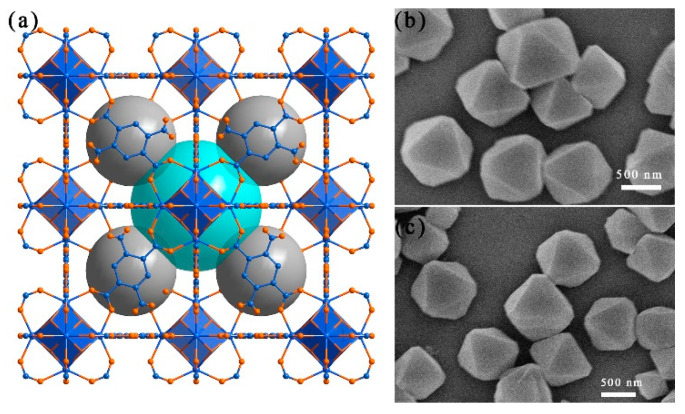
(**a**) Schematic illustration of the structure of UiO-66-(COOH)_2_. SEM morphology of (**b**) as-synthesized UiO-66-(COOH)_2_ and (**c**) Ag^+^@UiO-66-(COOH)_2_.

**Figure 2:**
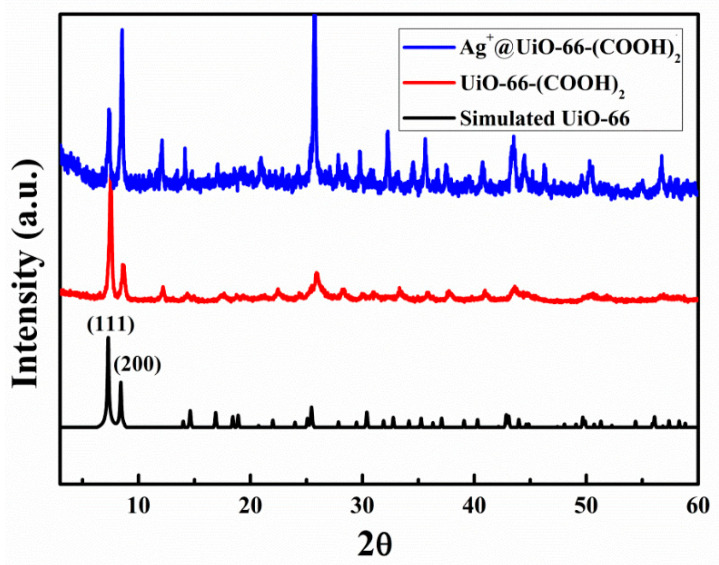
PXRD patterns of the simulated UiO-66, as-synthesized UiO-66-(COOH)_2_ and Ag^+^@UiO-66-(COOH)_2_.

The synthesis of UiO-66-(COOH)_2_ was realized by reflux reaction reported by our previous work [40], which exhibited high thermal and chemical stability which raised significant research interest in the past decade. This porous framework is built by Zr_6_-octahedra second building units that are linked by benzene-1,2,4,5-tetracarboxylic acid (H_4_btec) ligand, shaped into a cubic three-dimensional (3D) structure involving tetrahedral and octahedral cages (Figure 1a) [41]. Scanning electron microscopy (SEM) images were taken to reveal the morphology and size of the as-synthesized UiO-66-(COOH)_2_, which demonstrated that the MOF presents the octahedral structure with the size of 500–700 nm (Figure 1b). As shown in Figure 2, the power X-ray diffraction pattern of UiO-66 simulated from the single-crystal structure data features two peaks at 7.4° and 8.5°, respectively, corresponding to the crystal plane (111) and (200) [42]. The well-matched PXRD patterns of UiO-66-(COOH)_2_ to the simulated UiO-66 indicates an isostructural framework topology. As depicted in Figure 1a, only two carboxylates of the H_4_btec were coordinated to SBU, and the remaining two uncoordinated –COOH groups point to the pores of the framework. The presence of the uncoordinated –COOH groups was also evidenced by FT-IR spectrum. As shown in Figure S1, a strong band was observed at 1716 cm^−1^ which is attributed to the C═O stretching vibration of free –COOH groups. The permanent porosity of as-synthesized UiO-66-(COOH)_2_ was confirmed by N_2_ adsorption isotherm after guest removal, demonstrating the Brunauer–Emmett–Teller (BET) surface areas of 809.03 m^2^ g^−1^ (Figure S2). The uncoordinated –COOH groups and the permanent porosity indicated that UiO-66-(COOH)_2_ is a good candidate for post modification; therefore, we metalized UiO-66-(COOH)_2_ by the reaction of Ag^+^ ions with the uncoordinated –COOH groups in aqueous solution at 60 °C for one day. After the post-synthesis process, the PXRD of the as-obtained Ag^+^@UiO-66-(COOH)_2_ remained the same as UiO-66-(COOH)_2_ (Figure 2), indicating the maintenance of structure integrity during the modification process. The Ag^+^ loading level that was quantified by ICP-MS measurement shows the molar ratio of Zr:Ag is 1:1.62. Notably, after the post-synthesis process, the C=O stretching vibration of free –COOH groups almost disappeared at 1716 cm^−1^, giving a very direct proof of the interactions between the free –COOH groups and Ag^+^; furthermore, XPS deconvolution of the survey and Ag 3d spectrum of Ag^+^@UiO-66-(COOH)_2_ and AgNO_3_ is shown in Figure S3. It can be seen that the biding energy corresponding to the Ag 3d_3/2_ and 3d_5/2_ electronic orbit of Ag^+^@UiO-66-(COOH)_2_ is a little higher than those of AgNO_3_, indicating the coordination between Ag^+^ and –COOH groups; besides, the Ag^+^ incorporated framework remained at its original morphology, as revealed by the SEM images (Figure 1c). 

### 2.2. Iodide Adsorption

**Figure 3:**
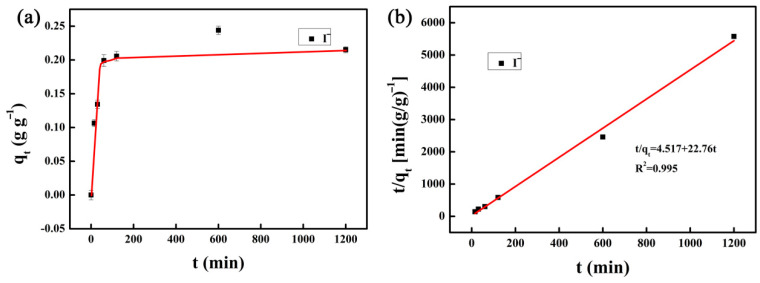
(**a**) The effect of contact time on the I^−^ adsorption on Ag^+^@UiO-66-(COOH)_2_; (**b**) pseudo-second-order plots of the time-dependent I^−^ adsorption.

On the basis of the high K_SP_ of AgI (8.3 × 10^−17^ mol^2^·L^−2^), the Ag^+^@UiO-66-(COOH)_2_ was expected to have a high affinity toward I^−^ in aqueous medium. To check this hypothesis, the removal efficiency Ag^+^@UiO-66-(COOH)_2_ on different initial concentrations of iodine was first explored. Afterwards, we prepared 0.5 g/L I^−^ solution in water and exposed sorbents to this solution while monitoring the concentration of I^−^ solution in water by ICP-MS. For comparison, the I^−^ adsorption ability of Ag^+^-free UiO-66-(COOH)_2_ was also investigated under the same experimental conditions. As shown in Figure 3a, more than 92% of equilibrium adsorption amounts were achieved within 60 min for I^−^. The adsorption to I^−^ could reach the equilibrium state within 20 h. The sorption kinetic was analyzed in terms of the pseudo-second-order rate equation. The linearized forms of the pseudo-second-order rate equation are presented in Equation (1).

(1) $$\frac{t}{q_{t}}=\frac{1}{k_{2}q_{e}^{2}}+\frac{t}{q_{e}},$$

where: *q_e_* and *q_t_* denote the amounts of iodide ions adsorbed at equilibrium *e* and at time *t*, respectively, and *k*_2_ (g/g min) is the second-order rate constant of adsorption.

The results and relevant parameters calculated from the fitting processes are shown in Figure 3b and listed in Table S1. The good fitting with the pseudo-second-order kinetic model suggests that the adsorption mechanism is chemisorption.

**Figure 4:**
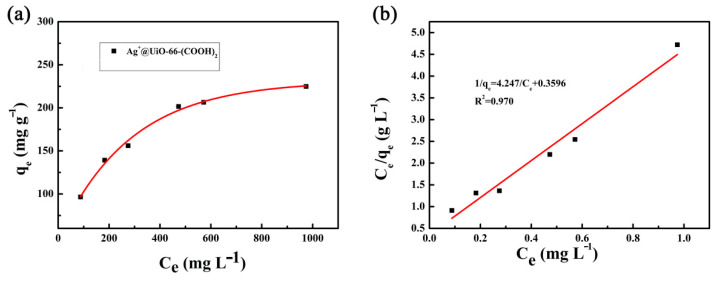
(**a**) Effect of initial concentration on the adsorption of I^−^ by Ag^+^@UiO-66-(COOH)_2_; (**b**) Langmuir isotherm models for the adsorption of I^−^.

To determine the adsorption isotherms, the initial I^−^ concentrations were set from 100 to 1000 mg/L and the results are shown in Figure 4a. Clearly, the amounts of I^−^ adsorbed on the Ag^+^@UiO-66-(COOH)_2_ increased with increasing initial concentration. The adsorption isotherms of I^−^ on the adsorbents were simulated using the Langmuir model, where its linear form can be described by Equation (2):

(2) $$\frac{C_{e}}{q_{e}}=\frac{1}{K_{L}q_{m}}+\frac{C_{e}}{q_{m}},$$

where *K_L_* (*L*/g) is the Langmuir adsorption coefficient and *q_m_* (g/g) is maximum adsorption capacity. Plots of 1/*q_e_* versus 1/*C_e_* of I^−^ for Ag^+^@UiO-66-(COOH)_2_ are shown in Figure 4b. The values of *K_L_*, *q_m_*, and R^2^ were obtained from the slope and intercept of linear correlation, as displayed in Table S2. From the Langmuir model, the maximum adsorption capacities of Ag^+^@UiO-66-(COOH)_2_ were determined to be 235.5 mg/g. In contrast, UiO-66-(COOH)_2_ can only adsorb 73.9 mg/g iodide under the same conditions as shown in Figure S4.

**Figure 5:**
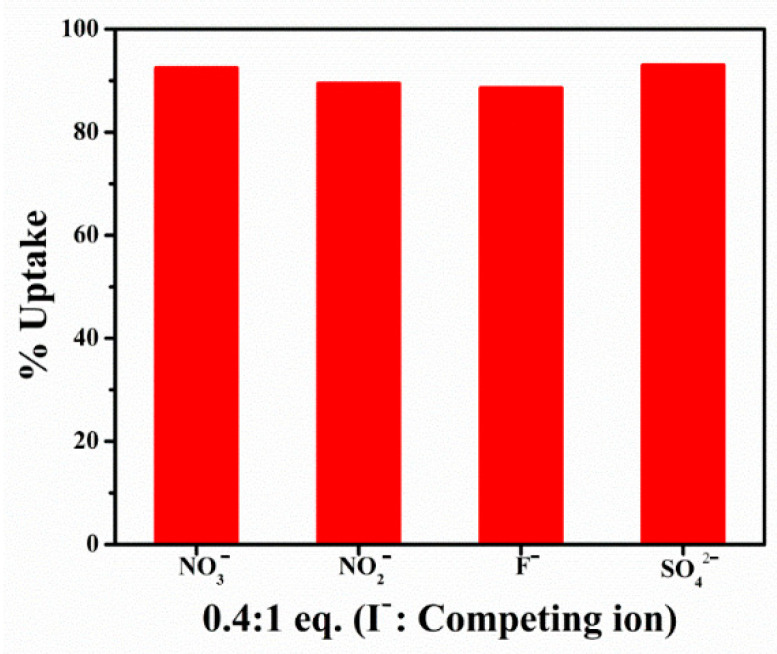
Relative uptake for I^−^ anions in a binary mixture of competing anions.

Furthermore, the complex exhibits outstanding adsorption behavior to I^−^ in diverse interference ions. Commonly, contaminated water contains several anions, including nitrate (NO_3_^−^), nitrite (NO_2_^−^), sulphate (SO_4_^2−^), and fluoride (F^−^), which may cause hindrance in the capture of I^−^. To check this, the removal of I^−^ was tested using a binary mixture of anions (0.5 g/L I^−^ and 1 × 10^−2^ M, other competing ions including NO_3_^−^, NO_2_^−^, SO_4_^2−^, and F^−^) by ion chromatography. At room temperature, the standard curve of iodide in aqueous solution was determined by the standard addition method as shown in Figure S5. The experiment revealed that 92.5%, 89.5%, 88.6%, and 93% I^−^ were the uptake percentages in the binary mixtures (Figure 5 and Figure S6), respectively, indicating exceptional binding for I^−^ of Ag^+^@UiO-66-(COOH)_2_, even in the presence of other competing ions. Some representative adsorbents and their performances are listed in Table S3, from which it can be seen that Ag^+^@UiO-66-(COOH)_2_ possesses outstanding adsorption capacity. 

### 2.3. Sensing Properties

The photoluminescent spectra of Ag^+^@UiO-66-(COOH)_2_ were examined in aqueous solution at room temperature. Upon the excitation of 381 nm, Ag^+^@UiO-66-(COOH)_2_ exhibited a broad emission peak with the center located at 497 nm, attributing to the ligand emission (Figure S7). As is well known, Ag^+^ is able to enhance the fluorescent emission of most emitters in aqueous solution [43]. Herein, the ligand-based emission intensity of Ag^+^@UiO-66-(COOH)_2_ was shown to be two times higher than UiO-66-(COOH)_2_ because of the sensitization of Ag^+^; however, due to the strong coordinating ability of Ag^+^ towards I^−^, it is expected that the Ag^+^ on the Ag^+^@UiO-66-(COOH)_2_ may be precipitated by I^−^, leading to a decease of the fluorescent intensity. 

**Figure 6:**
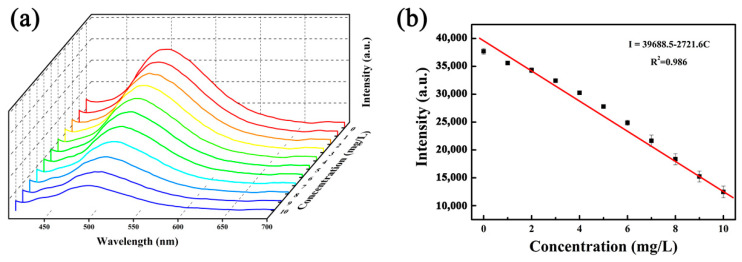
Emission spectra (**a**) and concentration dependence of the fluorescent intensity (**b**) of Ag^+^@UiO-66-(COOH)_2_ upon the addition of different concentrations of I^−^.

The above supposition was verified by the sensing experiments of Ag^+^@UiO-66-(COOH)_2_ and UiO-66-(COOH)_2_ towards I^−^ in aqueous solution. The fluorescent spectra changes of Ag^+^@UiO-66-(COOH)_2_ and UiO-66-(COOH)_2_ upon the addition of different concentration of Ag^+^ in aqueous solution were monitored by spectrometer. As shown in Figure 6a, with the increase in the I^−^ concentration (from 1 to 10 mg I^−^/L), the emission intensity of Ag^+^@UiO-66-(COOH)_2_ at 497 nm gradually decreases. Quantitatively, this phenomenon can be depicted using the linear equitation:

I_497_ = 39688.5 − 2721.6 × C(I^−^) (R^2^ = 0.986), (3)

The detection limit (LOD) was determined to be 0.58 ppm based on the formula of LOD = 3*δ*/S, where *δ* is the standard deviation for 30 replicating fluorescence measurements of blank solutions and *S* is the slope of the calibration curve. In contrast, the luminescent intensity of UiO-66-(COOH)_2_ shows no obvious change with the addition of I^−^ (Figure S8). 

**Figure 7:**
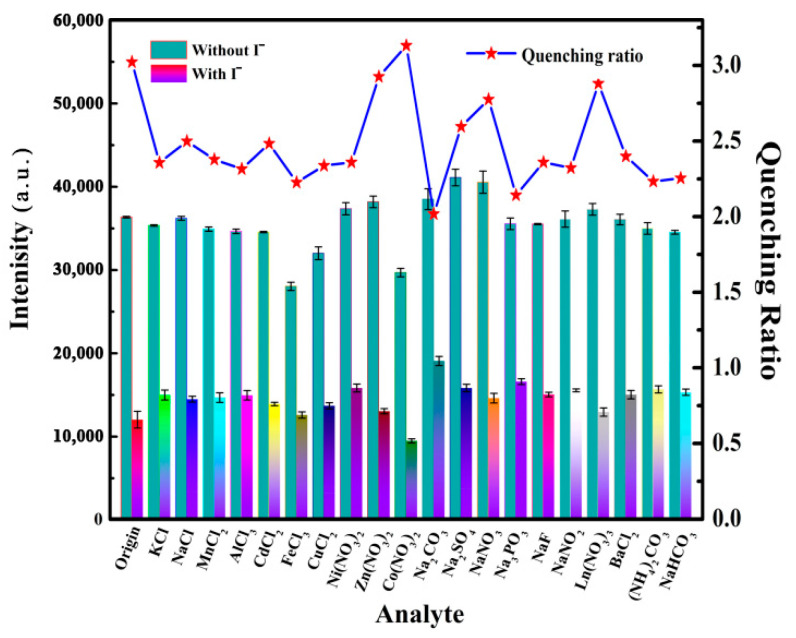
Changes of fluorescence intensity of Ag^+^UiO-66-(COOH)_2_ towards various analytes (1 × 10^−4^ M) before I^−^ addition (cyan column) and after I^−^ (10 mg/L) addition (multi-color column).

To verify the selectivity and anti-interference of this I^−^ probe, we tested the fluorescence response of Ag^+^@UiO-66-(COOH)_2_ to the environmentally relevant species in aqueous solution. The emission intensity of Ag^+^UiO-66-(COOH)_2_ is shown in Figure 7, where different interference ions (10^−4^ M) show slight fluctuations, especially in FeCl_3_ and Co(NO_3_)_2_ solutions; however, by adding 10 mg/L I^−^ ions into the solution, the luminescence of Ag^+^UiO-66-(COOH)_2_ in all solutions significantly quenched the process with a quenching ratio of approximately 2–3. These results demonstrate the great selectivity of I^−^ in the presence of a wide range of environmentally interfering species. Table S4 listed the representative fluorescent iodide probes, from which one can see that Ag^+^UiO-66-(COOH)_2_ shows comparable sensing ability with other MOF or small molecules sensors. 

## 3. Conclusions

In summary, an Ag^+^ incorporated nano-MOF Ag^+^UiO-66-(COOH)_2_ was prepared through post-synthetic method, and its adsorption and sensing performance towards I^−^ in aqueous solution was investigated. The successful incorporation of Ag^+^ on UiO-66-(COOH)_2_ was confirmed by FI-IR and XPS results, and the intact MOF structure after modification process was verified by XRD and SEM tests. Due to the high affinity of I^−^ to Ag^+^, Ag^+^UiO-66-(COOH)_2_ can effectively remove I^−^ in aqueous solution; moreover, after the precipitate of AgI, the fluorescent enhancement of Ag^+^ was reduced so that the luminescent of Ag^+^UiO-66-(COOH)_2_ was quenched, resulting in outstanding sensing ability toward I^−^. This work demonstrates that Ag^+^UiO-66-(COOH)_2_ is a good potential candidate for adsorption and sensing of iodide, which may raise research interests in the field of MOF-based adsorbents and sensors for the radioactive nuclide. 

## 4. Materials and Methods

### 4.1. Synthesis of UiO-66-(COOH)_2_

10 mmol 1,2,4,5-benzenetetracarboxylic acid (H_4_btec) and 10.4 mmol zirconium tetrachloride (ZrCl_4_) were dispersed in 60 mL distilled water in a round-bottom flask equipped with reflux condenser and magnetic stirrer; then, 40 mL acetic acid was added into the mixture at room temperature under vigorous stirring and heated at 100 °C for 24 h, obtaining a white powder product. Afterwards, the powder was washed with distilled water, anhydrous methanol, and acetone sequentially three times, respectively. During each washing process, the extract was decanted, and fresh water, methanol, or acetone was added every time. After the last washing process, the sample was dried in a vacuum oven at 80 °C to yield the final product. 

### 4.2. Preparation of Ag^+^@UiO-66-(COOH)_2_

Ag^+^@UiO-66-(COOH)_2_ was prepared by heating the mixture of UiO-66-(COOH)_2_ and AgNO_3_ in distilled water. Typically, the mixture of 0.1 g UiO-66-(COOH)_2_ and 0.17 g AgNO_3_ was mixed in distilled water and heated to 60 °C for 1 day; thereafter, the compound was isolated by centrifugation 8 min at 9000 rpm, and then washed three times with methanol followed by exchanging it with methanol for 1 day, followed by an activating process in a vacuum oven at 80 °C. 

### 4.3. Analytical Methods and Characterization

The morphology of the compound was characterized by scanning electron microscopy (SEM, HITACHI S-4800, Tokyo, Japan). X-ray photoelectron spectroscopy (XPS, kratos, Manchester, UK ) was carried out to verify the coordination reaction between the metal ions and MOF. The binding energy data were calibrated with reference to C 1s signal at 285 eV. Flourier transformation infrared (FT-IR) spectra were recorded on a Nicolet iS10 FT-IR spectrometer (Thermofisher, Waltham, MA, USA). XRD patterns were produced on the Shimadzu XRD 7000 diffractometer (Shimadzu, Tokyo, Japan) with Cu-Kα radiation at room temperature. The ion chromatography was carried out on an ICS-3000 ion chromatograph (Dionex, California, USA). Inductively coupled plasma mass spectrometry (ICP-MS) was performed on a Thermo Scientific XSERIES 2 ICP-MS system (Thermofisher, Waltham, MA, USA). Luminescence spectra for Ag^+^@UiO-66-(COOH)_2_ in aqueous solution were recorded on a Hitachi F4600 fluorescence spectrometer (Hitachi, Tokyo, Japan). 

### 4.4. Iodides Capture Studies

All the adsorption experiments were carried out in 10 mL centrifugal tubes at room temperature. 

Kinetic Studies. 1 mg UiO-66-(COOH)_2_ or Ag^+^@UiO-66-(COOH)_2_ were added into 10 mL solution containing 0.5 g I^−^/L in a centrifugal tube; then, the absorbance value of the supernatant solution was recorded at regular time intervals with the help of ICP-MS. 

Uptake Capacity Studies. 1 mg Ag^+^@UiO-66-(COOH)_2_ was kept in contact with 10 mL I^−^ solution bearing different amounts of I^−^ (100–1000 mg/L) for 24 h under stirring conditions. After 24 h, MOF was filtered out and the filtrate was analyzed by ICP-MS. 

Selectivity Studies. 1 mg Ag^+^@UiO-66-(COOH)_2_ was kept in contact with a binary solution containing 4 mL of I^−^ solution (0.5 g I^−^/L) and 4 mL each of various competing anions solution (1 × 10^−2^ M) for 24 h, including NO_3_^−^, NO_2_^−^, SO_4_^2−^, and F^−^; then, the concentration of I^−^ in the binary solution was detected by ion chromatograph.

### 4.5. Iodides Concentration Dependent Luminescence Spectra

0.5 mg of Ag^+^@UiO-66-(COOH)_2_ was dispersed in 1 mL of KI solution at the pH of 7.0 with different concentrations from 0 to 1 × 10^−6^ M and ultrasonic treatment for about 3 min to form a homogeneous suspension, and then the spectra were collected immediately. To investigate the influence of competing ions, 0.2 mg Ag^+^@UiO-66-(COOH)_2_ was dispersed into 1 mL of 1 × 10^−4^ BaCl_2_, La(NO_3_)_3_, Na_2_S_2_O_3_, Ce(NO_3_)_4_, AgNO_3_, NaNO_3_, NaCl, CaCl_2_, Zn(NO_3_)_2_, NaHCO_3_, CdCl_2_, NaF, MnCl_2_, and AlCl_3_ aqueous solution separately. 

## Data Availability

The data presented in this study are available on request from the corresponding authors.

## Supplementary Materials

The following supporting information can be downloaded at: https://www.mdpi.com/article/10.3390/molecules27238547/s1, Figure S1: FT-IR spectra of UiO-66-(COOH)_2_ and Ag^+^@UiO-66-(COOH)_2_; Figure S2: Survey XPS spectra of Ag^+^@UiO-66-(COOH)_2_ and AgNO_3_; Figure S3: Standard curve of iodide in aqueous solution by ion chromatography; Figure S4: Ion chromatography of binary mixture containing I^−^ and other competing anions; Figure S5: Excitation and emission spectra of Ag^+^@UiO-66-(COOH)_2_ in aqueous solution; Table S1: Kinetics parameters for I^−^ adsorption on Ag^+^@UiO-66-(COOH)_2_; Table S2: The Langmuir isotherm model parameters for I^−^ adsorption on Ag^+^@UiO-66-(COOH)_2_; Table S3: Comparison of the iodide adsorption in different adsorbents; Table S4: Comparison of the iodide sensing on different fluorescent probes [13,17,21,22,29,44,45,46,47,48,49,50,51].

## Sample Availability

Samples of the compounds are not available from the authors.
