# Supplementary material for: Highly Sensitive Adsorption and Detection of Iodide in Aqueous Solution by a Post-Synthesized Zirconium-Organic Framework
Source: Molecules. 2022 Dec 4;27(23):8547. doi: 10.3390/molecules27238547 (PMC9738272; doi:10.3390/molecules27238547)
Supplement: Supplementary file 1 [file molecules-27-08547-s001.zip › molecules-2038057-supplementary.pdf]

molecules

MDPI

Highly Sensitive Adsorption and Detection of Iodide in Aqueous Solution by a Post-Synthesized Zirconium-Organic Framework

Jun Zhang *,†, Shanli Yang †, Lang Shao, Yiming Ren, Jiaolai Jiang, Huaisheng Wang, Hao Tang, Hui Deng and Tifeng Xia *

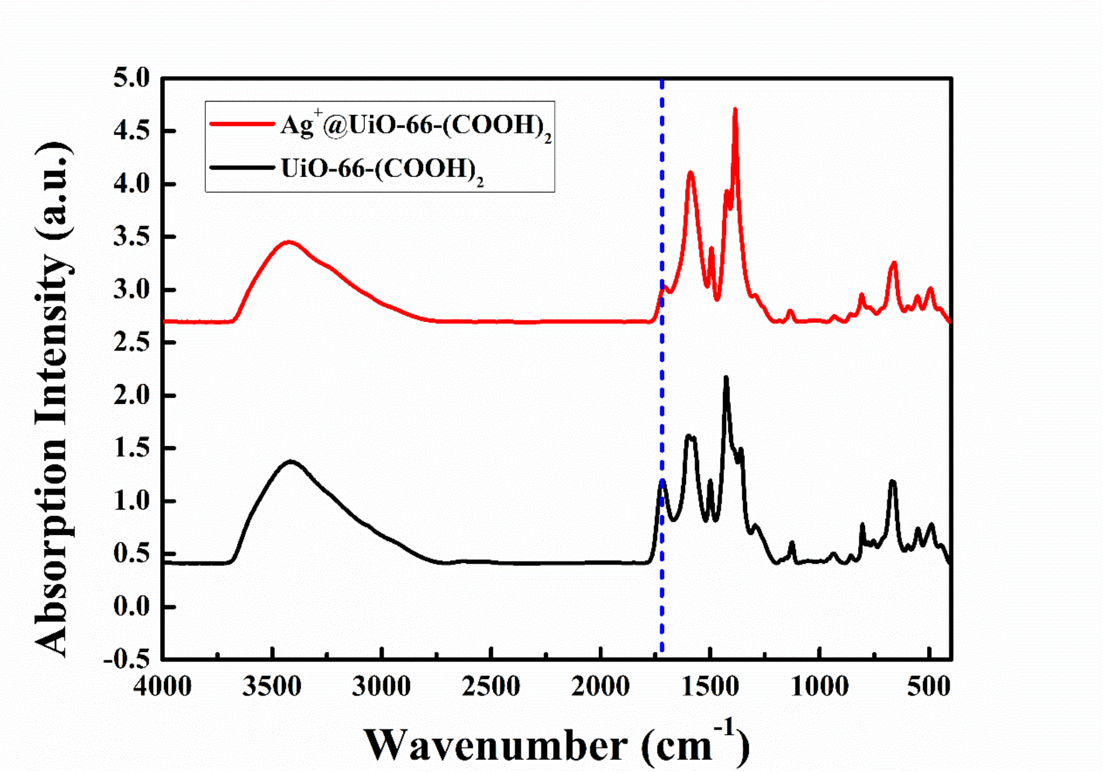

Figure S1. FT-IR sepctra of UiO-66-$(COOH)_2$ and Ag^+@UiO-66-$(COOH)_2$.

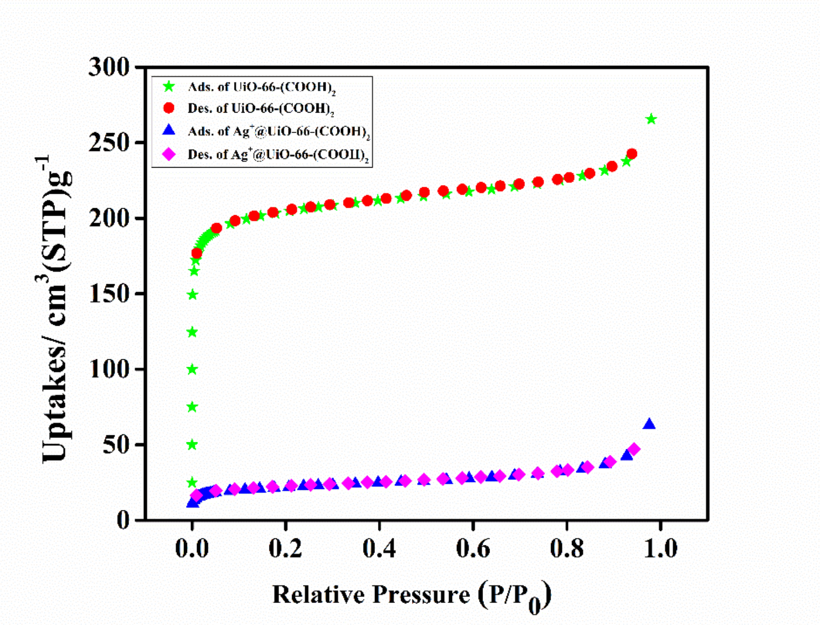

Figure S2. N_2 adsorption-desorption isotherms of UiO-66-$(COOH)_2$ and Ag^+@UiO-66-$(COOH)_2$ at 77 K.

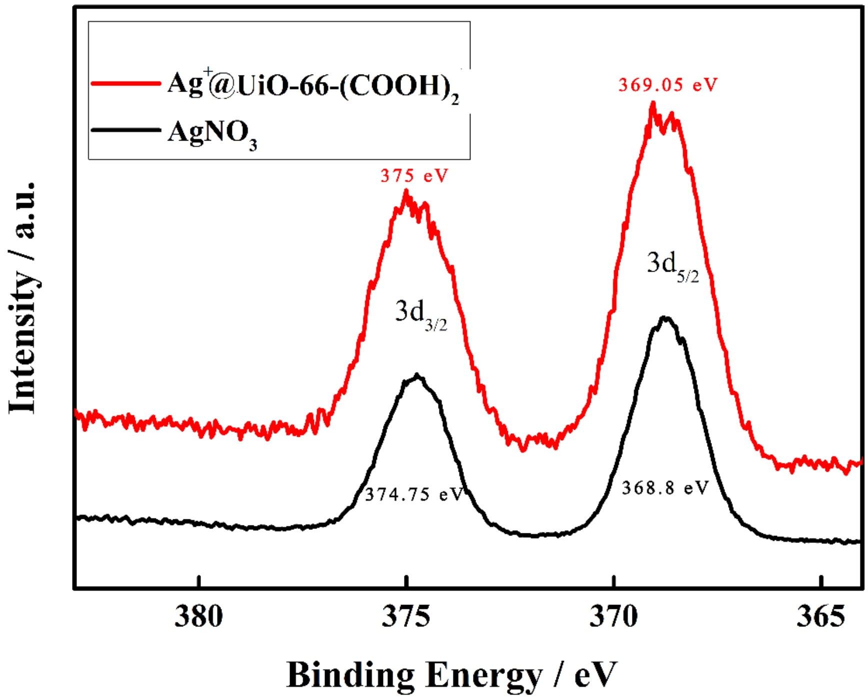

Figure S3. Survey XPS spectra of Ag^+@UiO-66-$(COOH)_2$ and $AgNO_3$.

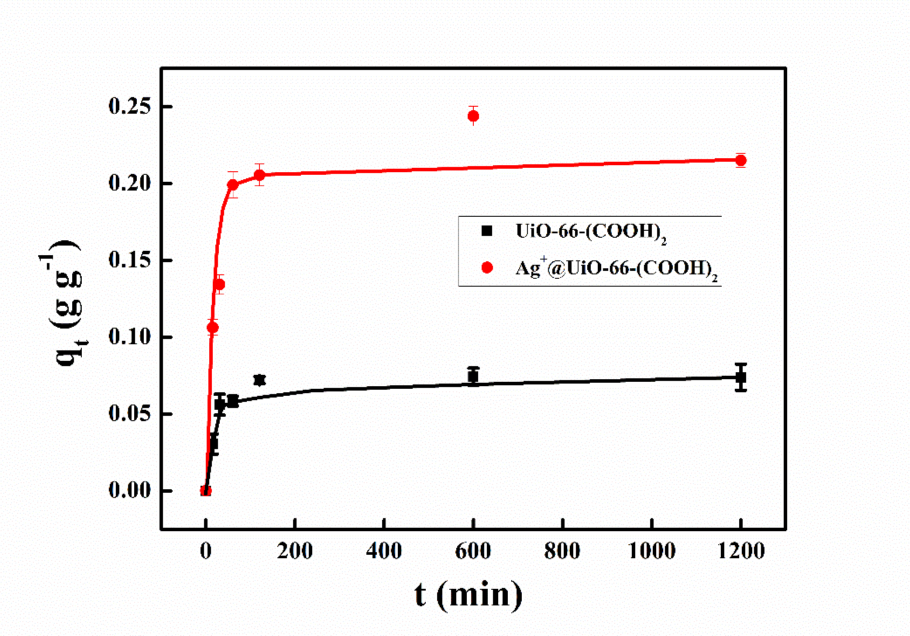

Figure S4. The effect of contact time on the I^- adsorption on UiO-66-$(COOH)_2$ and Ag^+@UiO-66-$(COOH)_2$ at room temperature.

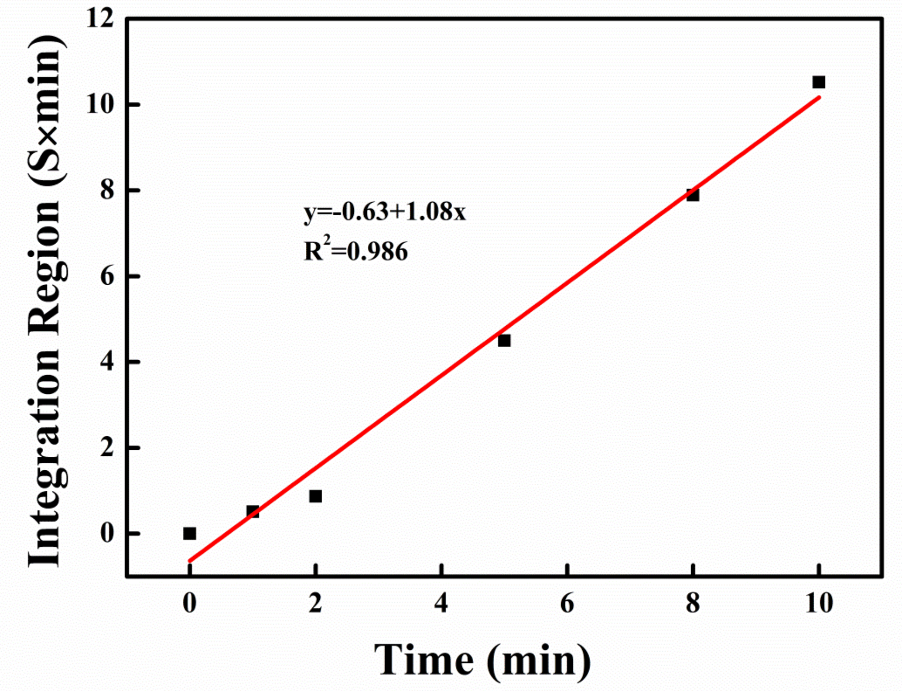

Figure S5. Standard curve of iodide in aqueous solution by ion chromatography.

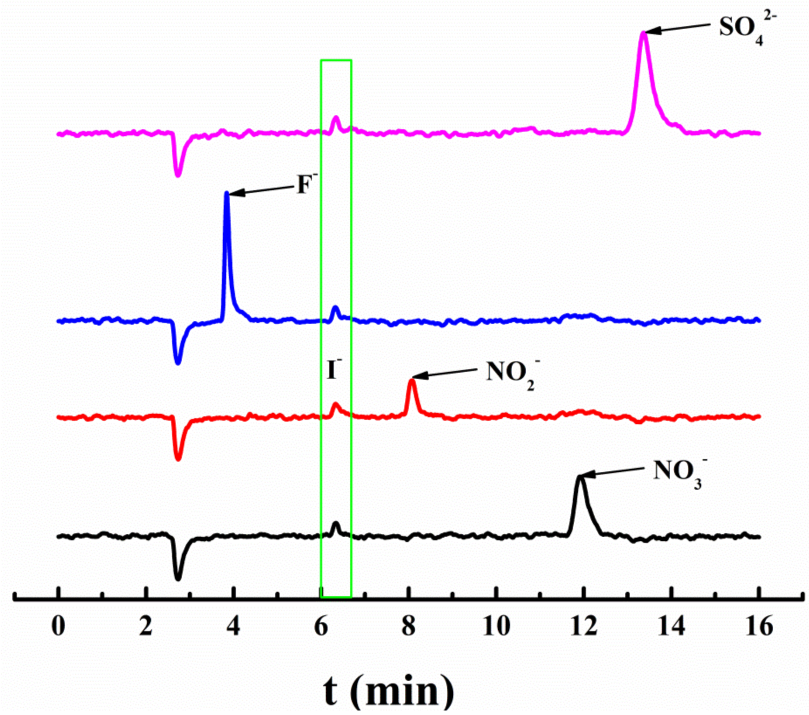

Figure S6. Ion chromatography of binary mixture containing I$^-$ and other competing anions.

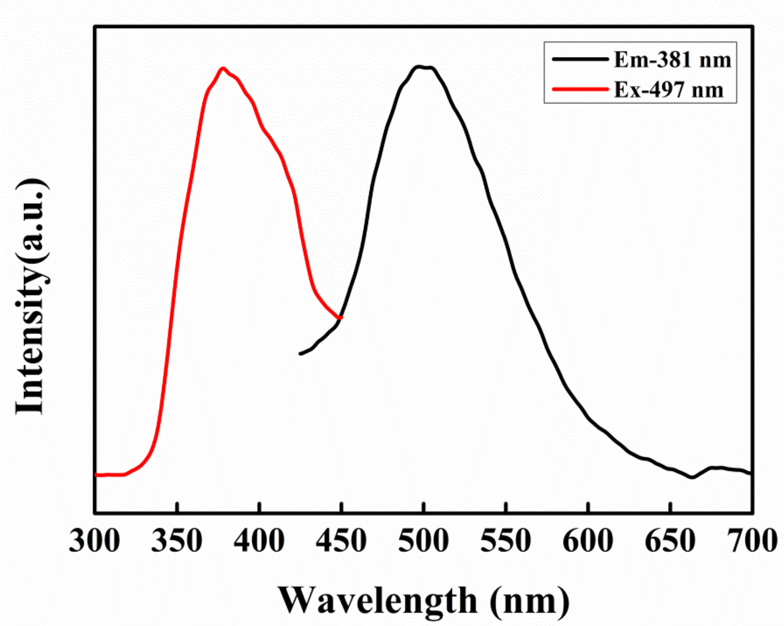

Figure S7. Excitation and emission spectra of Ag^+@UiO-66-$(COOH)_2$ in aqueous solution.

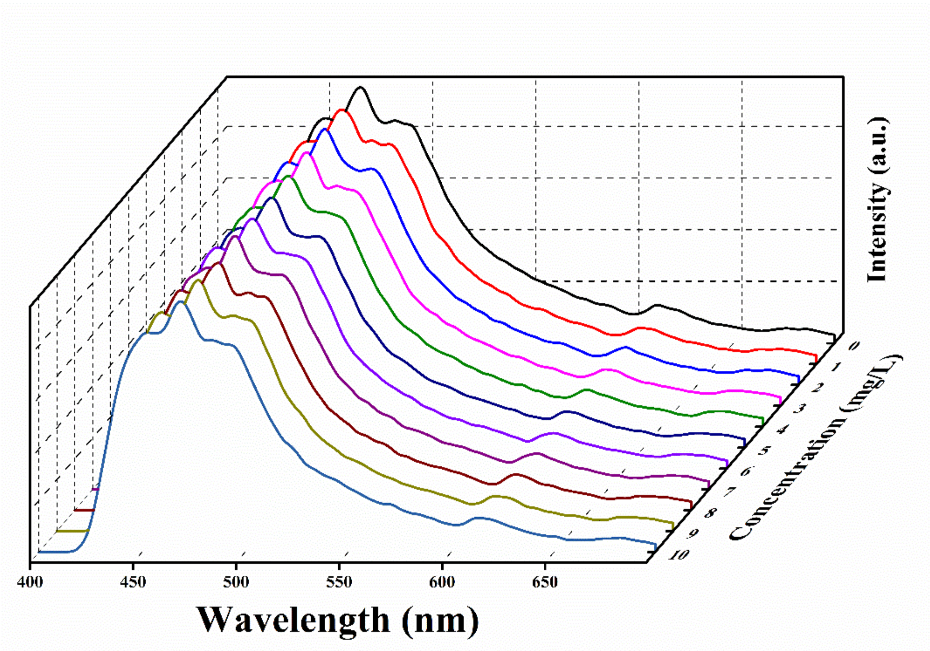

Figure S8. Emission spectra of UiO-66-$(COOH)_2$ upon the addition of different concentration of I^-.

Table S1. Kinetics parameters for I^- adsorption on Ag^+@UiO-66-$(COOH)_2$.

Concentration	k_2	Q_e	R^2
500 mg/L	0.898 (g/(g·min))	221.2 mg/g	0.995

Table S2. The Langmuir isotherm model parameters for I^- adsorption on Ag^+@UiO-66-$(COOH)_2$.

Materials	K_L	q_m	R^2
UiO-66-$(COOH)_2$@Ag^+	11.81 (L/g)	235.5 mg/g	0.970

Table S3. Comparison of the iodide adsorption in different adsorbents.

Adsorbent	Adsorption capacity (mg/g)	Equilibrium time	Ref.
MIL-101(Cr)-SO_3Ag	244.2	24 h	[1]
Nano Cu_2O/Cu-C	41.2	10 min	[2]
25%-Ag@MIL-101	2.14	2 h	[3]
Mg/Al/Bi MMO	202.2	12 h	[4]
1.0%-Ag@Cu_2O	25.38	2 h	[5]
Al_2O_3 carbon fiber	46.15	2 h	[6]
MIL-101(Cr)@Ag	57	10 min	[7]
**Ag^+@UiO-66-$(COOH)_2$**	**235.5**	**20 h**	**This work**

Table S4. Comparison of the iodide sensing on different fluorescent probes.

Probes	Linear range	LOD	Ref.
**Gold nanoclusters**	1-100 µM	0.056 ppm	[8]
**Thymine**	0.01-1000 µM	0.64 ppb	[9]
**Cz-TPM**	100-800 µM	1.10 ppm	[10]
**OTf**	0-50 µM	0.025 ppm	[11]
**Cd-MOF**	0-25 µM	0.088 ppm	[12]
**IPF**	1-10 µM	0.11 ppm	[13]
**Ag^+@UiO-66-$(COOH)_2$**	0.72-7.2 µM	0.58 ppm	This work

References

1. Zhao, X.; Han, X.; Li, Z.; Huang, H.; Liu, D.; Zhong, C. Enhanced removal of iodide from water induced by a metal-incorporated porous metal–organic framework. *Appl. Surf. Sci.* **2015**, *351*, 760–764.
2. Zhang X, Gu P, Li X, Zhang G, Efcient adsorption of radioactive iodide ion from simulated wastewater by nano Cu_2O/Cu modifed activated carbon. *Chem. Eng. J.* **2017**, 322, 129–139.
3. Mao P, Qi B, Liu Y, Zhao L, Jiao Y, Zhang Y, Jiang Z, Li Q, Wang J, Chen S, Yang Y, Ag-II doped MIL-101 and its adsorption of iodine with high speed in solution. *J. Solid State Chem.* **2016**, 237, 274–283.
4. Lee S-H, Takahashi Y, Selective immobilization of iodide onto a novel bismuth-impregnated layered mixed metal oxide: Batch and EXAFS studies. *J. Hazard. Mater.* **2020**, *384*, 121223.
5. Mao P, Liu Y, Jiao Y, Chen S, Yang Y, Enhanced uptake of iodide on Ag@Cu_2O nanoparticles. *Chemosphere*, **2016**, 164, 396–403.
6. Rong J, Zhao Z, Jing Z, Zhang T, Qiu F, Xu J, High-specifc surface area hierarchical Al2O3 carbon fber based on a waste paper fber template: preparation and adsorption for iodide ions. *J. Wood Chem. Technol.* **2017**, 37, 485–492.
7. Wan J, Li Y, Jiang Y, Yin Y, Silver-doped MIL-101(Cr) for rapid and effective capture of iodide in water environment: exploration on adsorption mechanism. *J. Radioanalytical Nuclear Chem.* **2021**, 328, 1041-1054.
8. Wang M, Wu Z, Yang J, Wang G, Wang H, Cai H, Au25(SG)18 as a fluorescent iodide sensor, *Nanoscale* **2012**, 4: 4087–4090.
9. Dai R, Wang X, Wang Z, Mu S, Liao J, Wen Y, Lv J, Huang K, Xiong X, A sensitive and label-free sensor for melamine and iodide by target-regulating the formation of G-quadruplex. *Microchem. J.* **2019**, 146, 592–599.
10. Dang Q, Wan H, Zhan X, Carbazolic porous framework with tetrahedral core for gas uptake and tandem detection of iodide and mercury, *ACS Appl. Mater. Interfaces* **2017**, 9, 21438–21446.
11. Salomón-Flores M, Hernández-Juárez C, Bazany-Rodríguez I, Barroso-Flores J, Martínez-Otero D, López-Arteaga R, Valdés-Martínez J, Dorazco-González A, Efficient fluorescent chemosensing of iodide based on a cationic meso-tetraarylporphyrin in pure water. *Sens. Actuators B* **2019**, 281, 462–470.
12. Singha D, Majee P, Mondal S, Mahata P, Luminescent cadmium based MOF as selective and sensitive iodide sensor in aqueous medium. *J. Photochem. Photobiol. A* **2018**, 356, 389–396.
13. Chen Z, Sun R, Feng S, Wang D, Liu H, Porosity-induced selective sensing of iodide in aqueous solution by a fluorescent imidazolium-based ionic porous framework, *ACS Appl. Mater. Interfaces* **2020**, 12, 11104–11114.
